# Supplementary material for: A Global Screen for Assembly State Changes of the Mitotic Proteome by SEC-SWATH-MS
Source: Cell Syst. 2020 Feb 26;10(2):133–155.e6. doi: 10.1016/j.cels.2020.01.001 (PMC7042714; doi:10.1016/j.cels.2020.01.001)

O60343 | TBCD4_HUMAN | TBC1D4 AS160 KIAA0603
Monomer MW [kDa]: 146.563 Monomer expected elution fraction: 37

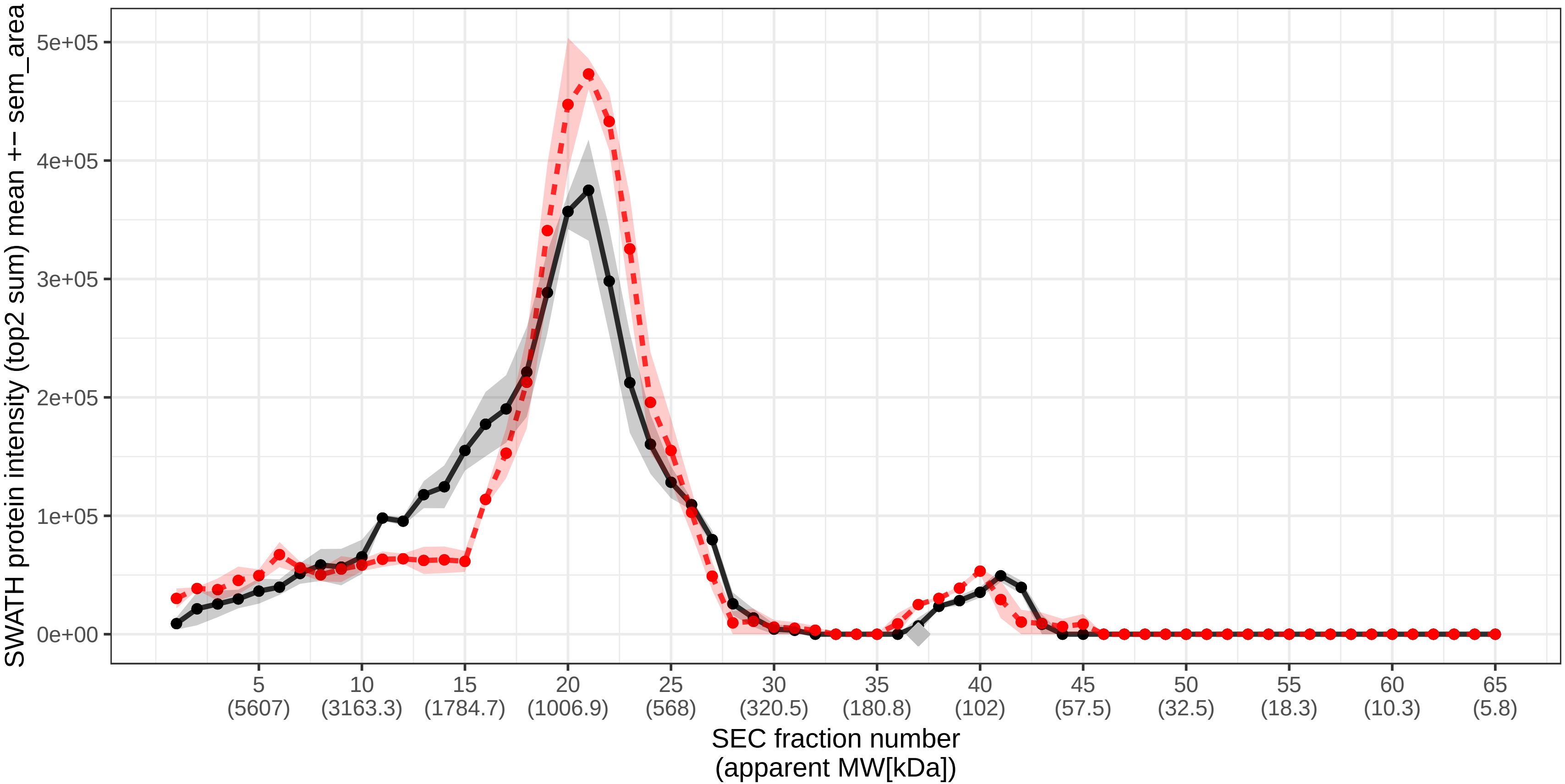

Supplement: Data S1. SEC-SWATH-MS Protein Chromatograms, Related to Figure 1 [file mmc6.zip › SECchrom_O60343_TBCD4_HUMAN_TBC1D4_AS160_KIAA0603.pdf]
